# The clinical and genetic factors for predicting the response to inhaled corticosteroids

**DOI:** 10.1186/2045-7022-3-S1-P15

**Published:** 2013-05-03

**Authors:** Hiroyuki Nagase, Naomi Tsurikisawa, Asae Kamiyama, Eiko Matsui, Naomi Kondo, Kazuo Akiyama, Ken Ohta

**Affiliations:** 1 Teikyo University School of Medicine, Department of Medicine, Japan; 2 National Hospital Organization Sagamihara National Hospital, Clinical Research Center, Japan; 3 Gifu University Graduate School of Medicine, Department of Pediatrics, Japan; 4 National Hospital Organization Tokyo National Hospital, Department of Respiratory Diseases, Japan

## Background

It has been reported that a part of asthma patients does not respond well to inhaled corticosteroids (ICS) and the benefit by long term treatment by high-dose ICS is limited in such patients. But the clinical or genetic factors for predicting ICS response have not been fully established especially in adults. The aim of this study is to establish the factor for predicting the response to ICS treatment.

## Method

Patients treated only by ICS for more than 6 months were enrolled and classified into responder group (R group, n=30) if FEV1 improvement ≥ 5% and non-responder group (NR group, n =40, FEV1 improvement<5%). The relationship between ICS response and pre-treatment clinical indices and 21 single nucleotide polymorphism (SNP) were retrospectively analyzed.

## Results

In R group, peripheral blood eosinophil % (R: NR = 7.5: 4.3%) and serum total IgE level (550.4: 497.1 U/ml) was significantly higher as compared to NR group. R group also showed significantly lower %VC (97.5: 114.8%), %FEV1.0 (77.9: 101.3%), FEV1.0% (67.5: 76.4%) and higher bronchial hyperresponsiveness. Bronchial reversibility test was available in 30 patients (R: n=11, NR: n=19) and reversibility was significantly higher in R group (15.9: 5.4%). By logistic multivariate analysis for those 30 patients, bronchial reversibility was significantly related to ICS response. SNP in IFN-γ R1 L467P was also related to ICS response. Post /Pre FEV1 ratio was significantly higher in homo/hetero group as compared to wild group (140.2 v.s 108.8%).

## Conclusion

Pre-treatment bronchial reversibility was identified as a prediction factor for ICS response. It has been reported that SNP in IFN-γR is associated with allergic diseases and serum IgE level and the SNP might be potentially related to ICS response.

